# The Increasing Effect of Interoception on Brain Frontal Responsiveness During a Socially Framed Motor Synchronization Task

**DOI:** 10.3389/fnhum.2022.834619

**Published:** 2022-05-20

**Authors:** Laura Angioletti, Michela Balconi

**Affiliations:** ^1^International Research Center for Cognitive Applied Neuroscience (IrcCAN), Università Cattolica del Sacro Cuore, Milan, Italy; ^2^Research Unit in Affective and Social Neuroscience, Department of Psychology, Università Cattolica del Sacro Cuore, Milan, Italy

**Keywords:** interoceptive attentiveness, motor task, fNIRS, PFC, synchronization, social joint task

## Abstract

This research explored the effect of explicit Interoceptive Attentiveness (IA) manipulation on hemodynamic brain correlates during a task involving interpersonal motor coordination framed with a social goal. Participants performed a task requiring interpersonal movement synchrony with and without a social framing in both explicit IA and control conditions. Functional Near-Infrared Spectroscopy (fNIRS) was used to record oxygenated (O2Hb) and deoxygenated hemoglobin (HHb) changes during the tasks. According to the results, the prefrontal cortex (PFC), which is involved in high-order social cognition and interpersonal relations processing, was more responsive when inducing the explicit focus (IA) on the breath during the socially framed motor task requiring synchronization, as indicated by increased O2Hb. In the absence of a broader social frame, this effect was not significant for the motor task. Overall, the present study suggests that when a joint task is performed and the individual focuses on his/her physiological body reactions, the brain hemodynamic correlates are “boosted” in neuroanatomical regions that support sustained attention, reorientation of attention, social responsiveness, and synchronization. Furthermore, the PFC responds significantly more as the person consciously focuses on physiological interoceptive correlates and performs a motor task requiring synchronization, particularly when the task is socially framed.

## Introduction

How does it feel to dance perfectly synchronized in front of an audience? How does it feel when you think about the sensations of your body while dancing or playing an instrument with another partner?

This ability to perceive, notice and pay attention to one’s internal body state, including visceral feelings, consists of interoception. Precisely, the focused attention to a particular interoceptive signal for a specified time interval, such as the breath, has been previously defined as Interoceptive Attentiveness (IA) (Schulz, 2016; Tsakiris and De Preester, 2018).

Conceived of as a deliberate focus on the breath that underpins practices of controlled breathing, IA has been demonstrated to have an enhancing effect on various cognitive and emotional functions like prolonged attentional focus, cognitive control, and awareness (Boyadzhieva and Kayhan, 2021; Weng et al., 2021), negative emotion modulation (Arch and Craske, 2006; Paul et al., 2007; Varga and Heck, 2017; Balconi and Angioletti, 2021b), and the ability to recover from high-stress levels (Grossman, 2011). Regarding the enhancing effect of IA, it is worth noting that IA is not a static dimension of interoception, but can be trained by specific types of interventions, like brief relaxation practices, mindfulness approaches, or even short controlled breathing sessions (Farb et al., 2013; Weng et al., 2021).

According to previous neuroimaging evidence, the IA lays its foundations on a network of cortical and subcortical structures, which included the posterior right and left insula, right claustrum, precentral gyrus, and medial frontal gyrus (Schulz, 2016). Recent findings revealed greater activation in several cortical regions for interoception including the insula and sensorimotor regions (postcentral gyrus, inferior parietal lobule, paracentral lobule, precentral gyrus, supplementary motor area) and occipital cortex, temporal cortex, anterior cingulate cortex, and lateral prefrontal regions (Stern et al., 2017). An activation of posterior insula was found in relation to cardioception, with a right-hemisphere dominance processing the non-verbal inputs. While an increase in neural activation at the level of the PFC has been found in relation to both the distribution of top-down attention and the processing of interoceptive information, probably mediated through the claustrum (Schulz, 2016).

A significant activation of these areas was not only found in relation to interoception, but supports other processes, including attention and motor control. In particular, the dorsolateral portion of the PFC is involved in orienting and sustaining focused attention on the target while controlling internal and external interferences (Kondo et al., 2004). It also helps to better sustain focus on the breath by improving the person’s awareness of when his or her mind wanders, allowing them to return their attention to the breath (Dickenson et al., 2013). In addition, this brain structure has been linked to social functions, by way of examples a fully conscious motor control and the adaptations to a changing rhythmic pattern (Stephan et al., 2002) or mutual cooperative interactions and interpersonal coordination (Balconi et al., 2017; Hu et al., 2021).

Nevertheless, to the best of our knowledge, there is no available evidence on how IA influences the prefrontal cortex (PFC) neural activity during interpersonal sensorimotor synchronization (interpersonal SMS), intended as movement among individuals that compels simultaneous occurrence, such as walking, drumming, playing an instrument, applause, or synchronized sports.

Interpersonal synchronization, in its widest terms, comprises a set of social communicative actions that encompasses joint attention, imitation, turn-taking, non-verbal social-communicative exchanges (Charman, 2011), and involves temporal and content synchronization (Delaherche et al., 2012). Interpersonal synchronization can occur either consciously when there is an explicit objective or unconsciously when the goal is absent. Motor synchronization is a subtype of interpersonal synchrony that focuses solely on non-verbal social-communicative exchanges and involves the synchronization of two people engaged in a social interaction (Fitzpatrick et al., 2017).

Former single-person synchronization studies showed the striato-thalamo-cortical system was involved in the timing process, and the coupling of motor and sensory areas engaged in rhythm perception. The cerebellum was critical to prediction and error correction, and activation of prefrontal and parietal areas was found in complex SMS tasks due to the high cognitive control demand (Repp and Su, 2013; Keller et al., 2014).

Regarding the social influence on synchronization performance, Dai et al. (2018) conducted a real-person joint-tapping hyperscanning experiment and found interpersonal SMS performance was better in a bidirectional than in unidirectional condition, suggesting bidirectional condition can be considered as more cooperative than unidirectional condition. Interestingly, two-person studies showed the right PFC of two interactive individuals exhibited synchronization during joint-tapping tasks (Cheng et al., 2015; Baker et al., 2016; Pan et al., 2017). It is, therefore, possible that even a simple motor task if openly socially framed can affect the neural activation of the participants engaged in the synchronization task.

Concerning the distinction between PFC and premotor cortices (PMC) activations, Bien et al. (2009) studied the functional separation between automatic and intentional imitation by using functional magnetic resonance imaging (fMRI): they found that frontal cortices transfer neural input about response inhibition to the PMC, which is engaged in automatic imitation. In line with the present understanding, the PFC seems to play a key role in social activities demanding synchrony (Sänger et al., 2011; Liu and Pelowski, 2014; Cheng et al., 2015), sustained attention (Khoe et al., 2020), and shared intentionality (operationalized as the joint attention to the stimulus with a mutual goal of problem-solving through interaction; Fishburn et al., 2018).

Notwithstanding, the influence of IA manipulation on tasks requiring interpersonal SMS has never been tested before. Furthermore, there is no observation of the effect of IA on synchronization when the task is not simply motor but has a declared social frame.

To investigate the potential positive impact that the manipulation of the attentional focus on the body could have on human social interactions can be an important research topic also in terms of promotion and intervention. In fact, an increased attentional focus on one’s body could promote the self-regulation of the individual and consequently obtain better performance also during a dynamic of social motor synchronization, such as sports, arts, but also in basic learning processes. IA is relevant for motor synchronization since interoceptive processes inform motor planning, making predictions about a partner’s movements, and motor coordination with the social partner (Farmer and Tsakiris, 2012). Also, previous studies suggested a link between controlled breathing and motor synchronization, stating the first plays a special role in mediating respiration-entrained brain synchrony enhancing motor activity (McKay et al., 2003) and synchrony in the motor cortex (Herrero et al., 2018).

Despite even basic interpersonal SMS, like for example rhythm tapping activity or walking, being shown to promote social bonds between partners (Wiltermuth and Heath, 2009; Vicaria and Dickens, 2016), as well as a self-reported sense of joint agency (Bolt et al., 2016), it remains still unexamined whether the PFC activation can be enhanced by consciously focused attention on the breath and by explicit social framing.

As a result, the aim of this work is to observe how intentional IA manipulation affects hemodynamic correlates of a motor synchronization task when it is or is not socially framed. In this experiment, the social framework applied to the motor task consisted only of explicitly requiring shared intentionality by the participants. Indeed, in the socially framed motor task, participants were told that they had to try to synchronize to develop greater teamwork skills. Therefore, the innovativeness and relevance of this task consist in maintaining the bidirectionality in interpersonal SMS and at the same time in emphasizing the mutual goal of the motor task.

In line with previous evidence supporting the positive impact of IA on cognitive processes (Weng et al., 2021), it was assumed that IA could magnify PFC correlates during the synchronized motor tasks, independently from the frame, compared to the control condition. Secondly, given the role of PFC in supporting social cognition and shared intentionality (Fishburn et al., 2018), an increased PFC activation for the socially framed motor task, compared to the simple motor task, is expected. Finally, given the involvement of PFC in promoting sustained attention (Khoe et al., 2020) and shared intentionality (Fishburn et al., 2018), we expect the PFC activation will be boosted in the socially framed motor task by the explicit IA condition.

## Methods

### Participants

For this fNIRS research, a total of eighteen healthy volunteers [14 females and four men; age mean (M) = 27.05; Standard Deviation (SD) = 3.18] were recruited from among university students with a non-probabilistic convenience sampling approach. Because the phenomenon under investigation is relatively new in the field of social neuroscience, and the literature does not provide systematic repeated evidence, previous references could not be used to estimate the extent of expected significant effects. As a result, we ran an *a priori* power analysis for repeated measures ANOVA and found that a total sample size of 17 (with alpha error probability = 0.05 and power 0.80) is the minimum for detecting a significant within effect or factor interaction [G*Power 3.1 software (Faul et al., 2007)].

Exclusion criteria included physiologic disorders such as chronic or acute pain, severe medical and chronic diseases, seizures, traumatic brain damage, pregnancy, previous meditation experience, and any mental or neurologic disorders. Participants were right-handed and with normal visual acuity. All subjects signed a written informed consent form prior to the experiment, and they were informed no compensation was provided. The Department of Psychology at the Catholic University of the Sacred Heart of Milan, Italy, gave its consent to the study, which was carried out in conformity with the Declaration of Helsinki.

### Study Procedure

Participants were introduced in a room with dimmed light and asked to sit next to an experimenter in charge of giving the experimental instructions and carrying out the synchronization tasks, together with the participant as a member of the dyad. Before starting with the experimental tasks, a 120-s resting baseline was recorded by using fNIRS.

Regarding IA manipulation, each participant performed the motor synchronization tasks in two distinct conditions: the explicit IA condition and a control condition (Balconi and Angioletti, 2021a,b). The explicit IA condition requested participants to focus on their body while executing the task, as it follows: *“During this task, we want you to focus your attention on your breath. As you execute the task, try to notice how you feel and whether there are any changes in your breath.”* Instead, in the control condition, participants were given instructions to complete the task, but no explicit request to concentrate on their interoceptive correlates was provided. The order of task execution was randomized and counterbalanced to prevent potential biases due to sequence effects. At the end of the four tasks, a set of manipulation checks were conducted for checking both their IA and social framing manipulations. Participants were asked to rate the attention they paid to themselves during the task on a Visual Analog Scale (VAS) with the following item “From 0 to 10, how much attention you paid to yourself during the task?,” the average score was for all the participants above 5 points (*M* = 7.88; SD = 1.62).

**FIGURE 1:**
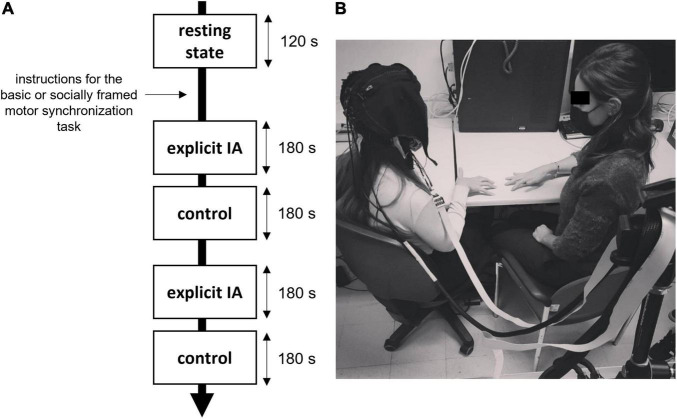
**(A,B)** Experiment setting and procedure. **(A)** Duration and timing of the motor tasks requiring synchronization, the instructions were provided both for the explicit IA and control conditions and for the type of the motor task (socially framed or not). **(B)** An example of an experimental setup with a fNIRS recording device and the experimenter performing the motor task.

The entire experiment took less than 40 min (Figure 1A).

### Motor Synchronization Tasks

The participants were required to perform a different version of the same motor synchronization task consisting of a simple finger movement task [modified version of the task adopted in a previous study (Jobbágy et al., 2005)]. For a total of 3 min, subjects were required to synchronize their finger movements with the experimenter sitting in front of them (Figure 1B).

For the finger movement task, the participants were told to position their dominant hand on the table in the prone position in front of the experimenter, with the fingers about one cm apart and elbows on the table. They were then asked to raise the fingers of their hand and tap the table with their little, ring, middle, index, and thumb. They were not told to perform the action at a specified tempo or raise their fingers as high as possible; instead, they were told to synchronize with the experimenter’s movement in front of them.

For the socially framed motor task, the task consisted of the same motor synchronization task previously described (i.e., the finger movement task), but it was socially framed by specifying that they need to synchronize during the motor task in order to develop greater teamwork skills. Our goal was to develop two experimental tasks: one in which subjects completed a motor task that supported shared intentionality and another in which the subjects completed the identical motor task, but the sharing of intention was not declared or emphasized. The order of the motor synchronization task execution was randomized and counterbalanced to prevent potential biases due to sequence effects (by computer-generated randomization).

At the end of the tasks, there was a debriefing phase in which participants declared their perceived sense of synchrony that was at the 98%. In the manipulation checks, the following item was included: *“From 0 to 10 how much do you think you were synchronized with your partner*?,” the average score was for all the participants above 5 points (*M* = 8.13; *SD* = 0.27).

### Functional Near Infrared Spectroscopy Montage

**FIGURE 2:**
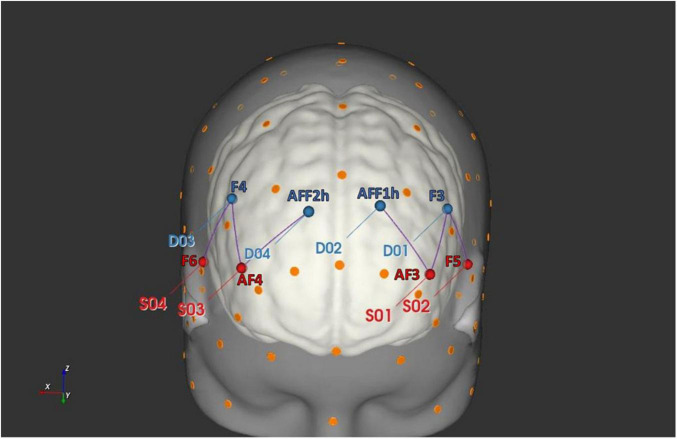
fNIRS head probe placement. The head rendering has been generated with the software NIRSite (NIRx Medical Technologies LLC) and it displays the position of the fNIRS sources (in red) and detectors (in blue).

For this study, oxygenated hemoglobin (O2Hb) and deoxygenated hemoglobin (HHb) variations were recorded by applying a six channel optodes matrix from a NIRScout System (NIRx Medical Technologies, LLC, Los Angeles, CA, United States). A fNIRS Cap was used to position four light sources/emitters and four detectors over the head in accordance with the standard international 10/5 system (Oostenveld and Praamstra, 2001). The emitter-detector distance was kept constant at 30 mm for consecutive optodes, and a near-infrared light with two wavelengths was used (760 and 850 nm). The fNIRS setup was consistent with a prior fNIRS research on IA (Balconi and Angioletti, 2021a) and in Figure 2 are reported the following six channels derived by optodes positioning: Ch1 (AF3-F3), Ch2 (AF3-AFF1h), Ch3 (F5-F3), that correspond to the left DLPFC, and Ch4 (AF4-F4), Ch5 (AF4-AFF2h), Ch6 (F6-F4) consistent with the right DLPFC (Brodmann Area, BA09). A probabilistic atlas implemented in the software fOLD [fNIRS Optodes’ Location Decider (Zimeo Morais et al., 2018)], based on the automated anatomical labeling atlas Brodmann (Rorden and Brett, 2000), was used for the correspondence between brain regions and channels.

### Hemodynamic Data Analysis

For fNIRS data acquisition and processing, the same procedural steps described in Balconi and Angioletti (2021a) were adopted. During the baseline (120 s) and the tasks, the hemodynamic signals (O2Hb and HHb) were acquired continuously with the NIRStar Acquisition Software. Each participant performed the two tasks in the two conditions one time. The average number of trials for each condition was no less than 45 for the 3 min, therefore 45 trials per each condition were considered. The signals from the six channels were collected at a sample rate of 6.25 Hz, then processed and analyzed by employing the nirsLAB software (v2014.05; NIRx Medical Technologies LLC, 15 Cherry Lane, Glen Head, NY, United States) based on their wavelength and position, yielding mmol*mm values related to the variations in O2Hb and HHb for each channel.

**FIGURE 3:**
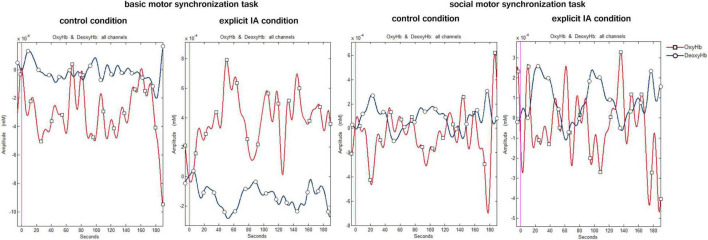
Hemodynamic signal time course under the four conditions. The figure shows the time course plots of O2Hb (red) and HHb signal (blue) when performing the following tasks: the basic motor synchronization task and the socially framed motor synchronization task during the control and explicit IA condition.

A digital band-pass filter was adopted to filter the raw data at 0.01–0.3 Hz (Balconi et al., 2015; Pinti et al., 2019). To detect noisy channels due to motion artifacts or amplitude changes raw time-series were visually inspected subject-by-subject both during the experimental phase and the signal analysis. 3% of the data was eliminated for artifacts. During this visual inspection channels with poor optical coupling and absence of ∼1 Hz heartbeat oscillations were excluded (Pinti et al., 2015). To preserve the frequency related to the task, a Mayer’s frequency was included in the filtering range which corrupts the optical cortical response estimation. In this regard, future research could adopt different methods to overcome this limitation. Moreover, a linear-phase FIR filter on respiration (0.3 Hz), that allows obtaining the symmetric-impulse-response, was used (Naseer and Hong, 2013; Naseer et al., 2014). For each channel, the average O2Hb and HHb were calculated and extracted for the two tasks performed in the two experimental conditions. Figure 3 shows the plots of the time course of O2Hb and HHb for all channels under the four conditions.

Following the data pre-processing, the average concentrations in the time series for each channel and individual were used to calculate the effect size in each condition. The following formula was adopted to calculate the effect sizes (Cohen’s d). They were calculated by dividing the difference between the baseline and trial means by the baseline standard deviation (SD): D = (m1-m2)/s, where m1 and m2 represent the baseline and trial mean concentration levels, respectively, and s represents the baseline SD. Unlike raw fNIRS data, which were originally relative values that could not be directly averaged across subjects or channels, normalized effect size data were averaged regardless of the unit because effect size is unaffected by differential pathlength factor (DPF). A preliminary step of the statistical analysis also included the single channels as a variable. However, since it was not significant anytime, the “Lateralization” as a variable created by grouping the frontal left (Ch1-Ch2-Ch3) and right (Ch4-Ch5-Ch6) homologous channels, was added to the successive statistical analysis to maintain a higher statistical power.

### Statistical Analysis

A set of repeated measures ANOVAs with independent within factors Condition (2: explicit IA, control) × *Task* (2: motor, social motor) × *Lateralization* (2: right, left) was applied to D-dependent fNIRS data (O2Hb and HHb concentration values). In the case of significant effects, pairwise comparisons were employed to check simple effects for significant interactions, and Bonferroni correction was utilized to decrease multiple comparisons possible biases. The degrees of freedom have been adjusted using Greenhouse-Geisser epsilon where suitable for all ANOVA tests. In addition, the normality of the data distribution was checked using the kurtosis and asymmetry indices. Partial eta squared (η^2^) indices were computed to determine the extent of statistically significant effects.

## Results

### Hemodynamic (O2Hb) Evidence

The statistical tests performed on D-dependent values for oxygenated and de-oxygenated concentrations yielded the outcomes reported below.

**FIGURE 4:**
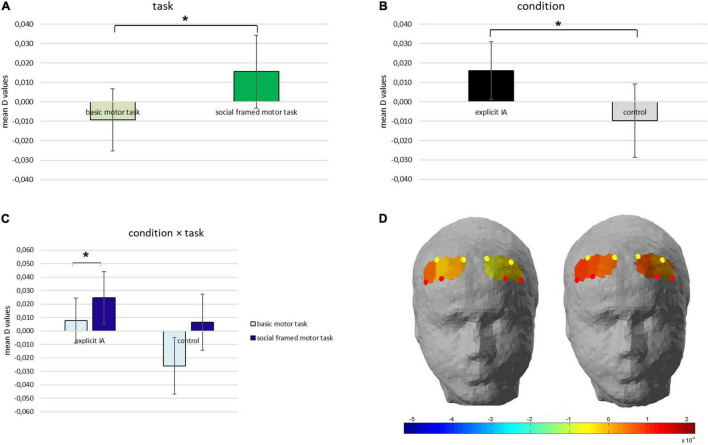
**(A–D)** Oxygenated hemoglobin (O2Hb) evidence. **(A)** The graph displays O2Hb modulation [**(D)** values] as a function of the Task, which is significantly increased for the social framed motor task compared to the basic motor task. **(B)** The bar chart shows significantly higher O2Hb values in the explicit IA confronted with the control condition. **(C)** The bar graph shows the significant interaction effect Condition × Task detected for the O2Hb values. **(D)** In the head renderings, it is represented the significant interaction effect for which the red color corresponds to the higher O2Hb values found in the socially framed motor task (right head) compared to the basic motor task (left head) in the explicit IA condition. All data are represented as mean ± SE; all asterisks mark statistically significant differences, with *p* ≤ 0.05.

A first significant main effect for the *Task* was detected for O2Hb values [*F*(1,17) = 8.04, *p* = 0.01, η*^2^* = 0.411], for which higher mean values were found in the socially framed motor task compared to the basic motor task (Figure 4A).

A second main effect was found for the *Condition* [*F*(1,17) = 6.43, *p* = 0.01, η*^2^* = 0.367], revealing significantly higher mean values of O2Hb in the PFC for the explicit IA condition compared to the control condition (Figure 4B).

Thirdly, a significant interaction effect *Condition* × *Task* was identified for O2Hb values [*F*(1,17) = 7.01, *p* = 0.01, η*^2^* = 0.397]. According to pairwise comparisons significant higher mean values were found in the socially framed motor task compared to the basic motor task in the explicit IA condition [*F*(1, 17) = 7.04, *p* = 0.01, η*^2^* = 0.402] (Figures 4C,D).

No significant effects were found for the lateralization factor.

Regarding the analysis conducted on the de-oxygenated D values, no significant results were detected.

## Discussion

This fNIRS research explored the effect of the explicit Interoceptive Attentiveness (IA) manipulation on hemodynamic brain correlates during a task involving interpersonal motor coordination framed with a social goal. According to the results, the PFC, which is involved in high-order social cognition and the processing of interpersonal relations, was more responsive, first of all, when inducing the explicit focus (IA) on the breath during the motor task requiring synchronization and secondly when the motor synchronization task was socially framed compared to the basic motor task, as indicated by increased O2Hb. Thirdly, in the explicit IA condition, this increase of O2Hb mean values in the PFC was found in the socially framed motor task compared to the basic motor task. In the absence of a broader social frame, this effect was not significant for the basic motor task.

Starting from the first evidence, increased O2Hb in the PFC was found when inducing the explicit focus (IA) on the breath during the motor task requiring synchronization. Former studies suggested the enhancing effect of IA on various cognitive and emotional functions like prolonged attentional focus, cognitive control, and awareness (Weng et al., 2021). This amplification effect seems to be mediated by the activation of the PFC, which, in tasks that require the interoceptive focus, is responsible for the distribution of top-down attention, the processing of interoceptive information (Schulz, 2016).

However, it is worth noting the task adopted in this study did not consist of an interoceptive task only, but also of a motor synchronization task. Before, the PFC has been linked to social functions, by way of examples a fully conscious motor control and the adaptations to a changing rhythmic pattern (Stephan et al., 2002) or mutual cooperative interactions and interpersonal coordination (Balconi et al., 2017; Hu et al., 2021). Therefore, it is plausible to state that IA plays a role in affecting the mental representation of the synergic task based on the PFC activation. In other words, the present study suggests that when a joint task is performed and the individual focuses on his/her physiological body reactions, the brain hemodynamic activation is significantly enhanced in neuroanatomical regions that support sustained attention, reorientation of attention, social responsiveness, and synchronization.

As a second effect, significantly higher mean O2Hb values were found in the socially framed motor task compared to the basic motor task. This effect could be deemed as a “social effect” that recalibrates the basic motor synchronization task.

In terms of the impact of social factors on synchronization performance, a previous hyperscanning study suggested that a more cooperative and bidirectional condition has a positive impact on interpersonal SMS performance (Dai et al., 2018). It is thus conceivable that even simple motor activity if openly socially framed, can have a distinct “social” impact on the PFC activation during a synchronization task. Notably, the PFC is involved in social activities demanding synchrony (Sänger et al., 2011; Liu and Pelowski, 2014; Cheng et al., 2015) and shared intentionality, meant as a shared focus on a task with the common purpose of problem-solving *via* the interaction (Fishburn et al., 2018). Therefore, this result is in line with our hypothesis that if there is a declared social purpose, then there is a massive effect of activating the PFC.

Finally, significantly higher mean O2Hb values were found in the socially framed motor task compared to the basic motor task in the explicit IA condition. Although basic interpersonal SMS tasks have also been shown to promote social bonds between partners (Wiltermuth and Heath, 2009; Vicaria and Dickens, 2016), as well as a self-reported sense of agency joint (Bolt et al., 2016), findings suggested that consciously focused attention on breathing in a condition with an explicit social framework could enhance neural activation of PFC areas that support shared intentionality, attentional focus, and high-order social processes. Interestingly, this effect was not found for the control condition.

Thus, we may suppose that combined effect of IA and a social frame, that is the condition in which the subjects are aware of their body changes, and they are mentally representing the social relevance of their motor synchronization, was related to the most significant PFC activation. In the other cases, the effect was absent or less significant in terms of PFC activation. Therefore, the innovative aspect of this study is that there a significant activation of the PFC as a person consciously focuses on its physiological interoceptive correlates and performs the socially framed motor synchronization task.

It should be noted that no significant effect was found for the lateralization factor in this study, perhaps suggesting that the effect of the combination of IA and a social purpose in this context does not recruit a significantly unbalanced hemispherical process. This result is interesting because it is partially in contrast with previous neuroscientific research has demonstrated activation of different portions of the right hemisphere during the execution of interoceptive attention/awareness (IAA) tasks (Farb et al., 2007; Zheng et al., 2019; Balconi and Angioletti, 2021a).

Thus, compared to previous studies, for the first time, the effect of IA manipulation on synchronization was observed not only when the task is simply a motor task, but also when it is framed with a social purpose requiring shared intentionality. It is as if the focus on yourself and your breath while dancing in a synchronized manner with a partner, in order to demonstrate excellent performance in front of an audience, activates more the neural basis that supports the performance.

Some relevant applications can be suggested for the social, clinical domain and, potentially, for rehabilitation contexts. For example: in socially mediated sports disciplines, through practices that involve self-perception, it could be possible to strengthen the sense of belonging and teamwork between the players. Or, in rehabilitation, the sense of awareness of one’s own body reactivity and the impact it has at the level of the central nervous system could be a target of therapy, in order to manage consciously (when possible) mental or neurophysiological deficits, developing a joint relationship with tutors or clinicians.

The current study holds potential for innovation, nonetheless, there are the following caveats to consider. First, this work used a simplest type of interpersonal SMS requiring finger tapping and not more complex motor synchronized activities (like dancing or playing an instrument). Complex situations, such as ecological tasks, multi-person groups, or tempo changes should be investigated by further studies, paying attention to controlling the amount of cognitive load. However, the present study used the simplest model of interpersonal SMS as an example to evaluate the effects of the explicit IA manipulation and the social frame on the PFC.

Secondly, the social framework applied to the motor task consisted only of explicitly requiring shared intentionality to the participants (operationalized as the instruction to synchronize to develop greater teamwork skills). However, distinct instructions, less artificial and contextually closest to the synchronization task could be given to the participants to increase the ecological validity of the results. For example, in the case of a motor synchronization task that requires participants to playing an instrument, the social framed condition might request them to synchronize to develop their duet skills. In this way, we could observe the effect of the interoceptive manipulation on the neural responses connected to a more ecological social framing condition.

Thirdly, fNIRS was only used on the PFC and not on the complete brain, including somatosensory cortical areas and subcortical structures (Balconi and Molteni, 2016), such as PMC and the claustrum. These two structures play a role in intentional imitation (Bien et al., 2009) and interoceptive processing (Schulz, 2016) respectively, thus their role deserves further deepening by future works. On a methodological level, prospective research could also implement fNIRS layouts that include short channels, as method that can help to eliminate non-specific hemodynamic trends and minimize noise (Brigadoi and Cooper, 2015).

In order to generalize these findings, the sample size should be enlarged by recruiting more participants, and gender should be balanced in future studies.

Also, specific manipulation checks to make sure the participants only focused on their breath and not on other modalities (e.g., skin temperature or heartbeat) could be added in future studies. Otherwise, it will be required to control the voluntary component of the respiratory rate, which could affect the overall outcomes of this research, and to examine the differences between tasks that require a focus on breathing and tasks that require a focus on cardiac activity.

To sum up, future research should consider exploring the influence of IA and social framing in more realistic situations such as dancing and playing an instrument. Most importantly, on a methodological level, collecting the brain activity of a dyad while executing these activities could be valuable (for instance by applying the hyperscanning paradigm) for grasping the interbrain coherence of the partners and observing the neural dynamic of the two subjects synchronizing with each other, rather than the responses of the single subject synchronizing with the experimenter. This would allow us to explore how the increasing effect of interoception on the PFC functions in the dyad at the neural level during a socially framed motor synchronization task.

## Data Availability Statement

The raw data supporting the conclusions of this article will be made available by the authors, without undue reservation.

## Ethics Statement

The studies involving human participants were reviewed and approved by Ethic committee of the Department of Psychology, Catholic University of the Sacred Heart, Milan, Italy. The patients/participants provided their written informed consent to participate in this study. Written informed consent was obtained from the individual(s) for the publication of any potentially identifiable images or data included in this article.

## Author Contributions

MB and LA contributed to the conception and design of the study, wrote the first draft, and contributed to the manuscript revision. Both authors read and approved the submitted version.

## Conflict of Interest

The authors declare that the research was conducted in the absence of any commercial or financial relationships that could be construed as a potential conflict of interest.

## Publisher’s Note

All claims expressed in this article are solely those of the authors and do not necessarily represent those of their affiliated organizations, or those of the publisher, the editors and the reviewers. Any product that may be evaluated in this article, or claim that may be made by its manufacturer, is not guaranteed or endorsed by the publisher.
